# Targeted Therapy Database (TTD): A Model to Match Patient's Molecular Profile with Current Knowledge on Cancer Biology

**DOI:** 10.1371/journal.pone.0011965

**Published:** 2010-08-10

**Authors:** Simone Mocellin, Jeff Shrager, Richard Scolyer, Sandro Pasquali, Daunia Verdi, Francesco M. Marincola, Marta Briarava, Randy Gobbel, Carlo Rossi, Donato Nitti

**Affiliations:** 1 Clinica Chirurgica Generale 2, Department of Oncological and Surgical Sciences, University of Padova, Padova, Italy; 2 Symbolic Systems Program, Stanford University, and CollabRx, Inc., Palo Alto, California, United States of America; 3 Tissue Pathology and Diagnostic Oncology, Royal Prince Alfred Hospital, Sydney, Australia; 4 Melanoma Institute Australia, Sydney, Australia; 5 Discipline of Pathology, The University of Sydney, Sydney, Australia; 6 Infectious Disease and Immunogenetics Section, Department of Transfusion Medicine, Clinical Center, and Center for Human Immunology, National Institutes of Health, Bethesda, Maryland, United States of America; Faculdade de Medicina, Universidade de São Paulo, Brazil

## Abstract

**Background:**

The efficacy of current anticancer treatments is far from satisfactory and many patients still die of their disease. A general agreement exists on the urgency of developing molecularly targeted therapies, although their implementation in the clinical setting is in its infancy. In fact, despite the wealth of preclinical studies addressing these issues, the difficulty of testing each targeted therapy hypothesis in the clinical arena represents an intrinsic obstacle. As a consequence, we are witnessing a paradoxical situation where most hypotheses about the molecular and cellular biology of cancer remain clinically untested and therefore do not translate into a therapeutic benefit for patients.

**Objective:**

To present a computational method aimed to comprehensively exploit the scientific knowledge in order to foster the development of personalized cancer treatment by matching the patient's molecular profile with the available evidence on targeted therapy.

**Methods:**

To this aim we focused on melanoma, an increasingly diagnosed malignancy for which the need for novel therapeutic approaches is paradigmatic since no effective treatment is available in the advanced setting. Relevant data were manually extracted from peer-reviewed full-text original articles describing any type of anti-melanoma targeted therapy tested in any type of experimental or clinical model. To this purpose, Medline, Embase, Cancerlit and the Cochrane databases were searched.

**Results and Conclusions:**

We created a manually annotated database (Targeted Therapy Database, TTD) where the relevant data are gathered in a formal representation that can be computationally analyzed. Dedicated algorithms were set up for the identification of the prevalent therapeutic hypotheses based on the available evidence and for ranking treatments based on the molecular profile of individual patients. In this essay we describe the principles and computational algorithms of an original method developed to fully exploit the available knowledge on cancer biology with the ultimate goal of fruitfully driving both preclinical and clinical research on anticancer targeted therapy. In the light of its theoretical nature, the prediction performance of this model must be validated before it can be implemented in the clinical setting.

## Introduction

### Targeted Therapy in Cancer Medicine

Cancer represents the third leading cause of death worldwide and the second in Western countries [1], [2]. Early diagnosis continues to offer the best chance of cure for most tumor types. The efficacy of currently available anticancer treatments are far from satisfactory in the advanced/metastatic setting where most patients succumb to their disease. General agreement exists regarding the urgency of developing molecularly targeted therapies, their implementation in the clinical setting being in its infancy [3], [4], [5], [6], [7], [8].

The term “targeted therapy” includes all those approaches that aim to tailor the therapy to the patient (or cohort of patients) based on specific molecular features of the disease- and/or patient [3], [7], [9], [10], [11], [12], [13], [14]. The ultimate goal is obviously to maximize the therapeutic efficacy while minimizing the toxicity, that is, increasing the “therapeutic index”. In cancer medicine, tumor-specific molecular derangements (e.g., gene mutation or protein overactivation), are the ideal targets for therapeutic strategies aimed to kill malignant cells while sparing normal cells. Furthermore, patient-specific molecular features such as polymorphisms of detoxifying enzymes can affect the metabolism of anticancer drugs and thus can play a role in both efficacy and toxicity profiles. According to these principles, personalized targeted therapy includes not only the development and clinical implementation of “smart” drugs (i.e., agents that target tumor-specific molecular derangements), but also the identification of the patient molecular profile that maximizes the therapeutic index of “conventional” chemotherapeutics.

Therefore, the two mainstreams of research in the field of targeted anticancer therapy can be summarized as follows:

- to develop novel therapeutic agents based on the molecular “Achilles' heel(s)” of malignant cells, which usually implies the selection of patients bearing a cancer that harbors that specific molecular derangement;
- to identify biomarker(s) predictive of tumor responsiveness based on the molecular characteristics of both the patient and the tumor; this approach, ultimately, would lead to administer conventional and/or targeted drugs only to patients with the greatest likelihood of responding and the least likelihood of suffering from side effects.

Research on anticancer targeted therapy has made several advances; a number of “smart” approaches have now reached the clinical phase of experimentation and some of them have been approved for the routine treatment of patients affected by specific types of cancer [5], [8], [15], [16], [17], [18], [19], [20], [21], [22]. Nevertheless, there is general agreement that most work is still to be done before we can state that targeted therapy is the standard of care for cancer. In this regard, the most important hurdles appear the following: 1) elucidation of the molecular pathways governing disease development and progression has provided investigators with numerous potential new therapeutic targets, but has at the same time exponentially increased the number of variables that must be taken into account when designing new drugs and trials; 2) the ever growing amount of information generated by the scientific community stands in striking contrast to the parallel lack of publicly available bioinformatic tools capable of integrating data and knowledge in a rationally organized, biologically informative and therapeutically oriented manner, which would maximize the likelihood of finding the shortest path to effective cancer treatments; 3) therapy personalization requires the study of molecular profiles on a single-patient basis, which requires the availability of huge computable biological databanks; a formidable corollary issue is that data sharing implies the compatibility of different technological platforms used around the world by different investigators (as exemplified by the CaBig project, https://cabig.nci.nih.gov); 4) the costs for the development and the production of “smart” drugs may pose problems of expenses that cannot be sustained by either public or private research institutions or even by national health care systems.

Overall, despite the wealth of preclinical studies addressing the issue of targeted anticancer therapy, the complexity of testing each preclinical hypothesis in the clinical arena represents an intrinsic obstacle. As a consequence, the gap is widening between the pace of discovery in the field of cancer biology and the improvements in therapeutic benefit for patients. In particular, the scientific community has only recently acknowledged that the lack of tools for the systematic and therapy-oriented collection of the biomedical data may ultimately cause an enormous and paradoxically unethical waste of information [23], [24], [25], [26], [27], [28], [29], [30], [31], [32].

The creation of an open-access repository for the storage and the analysis of data on targeted therapy is a relatively feasible step towards the full exploitation of the information produced by the scientific community. Although some attempts have been made in this direction [33], [34], [35], [36], [37], [38], [39], no disease-specific project exists to systematically collect and comprehensively exploit scientific data for the therapeutic management of patients.

The objective of the present project is to create a manually annotated database where the relevant data are gathered in a formal representation that can be computationally analyzed for the identification of therapeutic hypotheses based on the available evidence and for ranking treatments based on the molecular profile of single patients.

To this aim we focused on melanoma, an increasingly diagnosed malignancy for which there is an urgent need to develop novel therapeutic approaches since no effective treatment is available, especially in the advanced setting.

### The case of melanoma

Although cutaneous malignant melanoma is the least common form of skin cancer, it accounts for 75% of skin cancer deaths [2], [40], [41], [42], [43]. During most of the twentieth century, the incidence of melanoma in populations of European origin rose faster than any other solid cancer, barring lung cancer. An estimated 160,000 new cases and 41,000 deaths were reported worldwide in 2002. In the United States, the American Cancer Society reported approximately 60,000 new cases of melanoma (with an estimated lifetime risk of 1 in 49 for men and 1 in 73 for women), leading to an expected 8,110 deaths in 2007. In comparison, the incidence in 2001 was approximately 47,700 new cases. This underscores that melanoma is an important and growing public health concern.

The therapeutic management of cutaneous melanoma is one of the most challenging issues for oncologists [40], [41], [42]. Because melanoma is among the solid malignancies most refractory to medical therapy, early diagnosis coupled with surgical removal of the primary tumor is virtually the only curative approach currently available. For metastatic melanoma, no conventional or molecularly targeted drug is better than dacarbazine (DTIC); however, there is no convincing evidence that DTIC is better than best supportive care [44], [45], [46].

In patients with high-risk melanoma, ie, with American Joint Committee on Cancer (AJCC) TNM stage II (T2-4 N0 M0) and III (Tany N+ M0) disease the rate of disease recurrence ranges between 20% to 60%, with 5-year overall survival (OS) varying between 45% and 70% [47]. The only agent currently approved treatment for such patients after apparently radical surgery (ie, adjuvant setting) is interferon (IFN) alpha [48]: according to the most recent meta-analysis published on this subject, the use of IFN-alpha reduces the risk of death by about 10% [49].

Overall, it is clear the urgency of accelerating the pace at which novel, effective therapeutic options can be offered to patients affected with melanoma.

From a translational perspective, one way of maximizing the practical usefulness of the available scientific evidence would be to share the knowledge and organize it in a computationally oriented fashion: ultimately, this would allow to comprehensively utilize both clinical and preclinical information on targeted therapy for the therapeutic management of patients.

In 2007 we have started an initiative in this direction by launching the Melanoma Molecular Map Project (MMMP, http://www.mmmp.org), an open-access website dedicated to the systematic collection of scientific information on melanoma biology and treatment [50]. The MMMP website, which presently collects more than 4,000 records distributed in seven interconnected databases, currently ranks first as “melanoma database” in the Google search engine.

This essay describes the main features of a newly implemented MMMP database called Targeted Therapy Database (TTD), which specifically focuses on the available scientific information that can be exploited to promote the development of personalized treatments for patients affected with melanoma.

## Methods

### The Targeted Therapy Database

The Targeted Therapy Database (TTD) is a systematic collection of the scientific knowledge regarding the development of targeted therapy for melanoma. A copy of the database is available as an open-access file in the MMMP website (http://www.mmmp.org).

This database is intended to gather in a standardized and computationally oriented fashion the published evidence on the molecular features that have been so far investigated to develop melanoma-specific therapies.

The TTD can be queried for the following purposes:

1. To provide both basic researchers and clinical investigators with an unprecedented synopsis of the available scientific literature regarding the targeted therapy of melanoma;
2. To obtain summaries of the current evidence about the relationship between single molecules (or set of molecules) and the efficacy (or toxicity) of a given therapeutic agent (or set of therapeutic agents); summaries regarding the synergisms between drugs (conventional and/or targeted drugs) can also be obtained;
3. To match the patient (cancer) molecular profile with the available scientific evidence about the targeted therapy of melanoma, thus developing a drug ranking system for the personalized treatment of melanoma.

As such, the information collected in the TTD will provide an overall picture of the data produced by the scientific community with regard to anti-melanoma targeted therapy, which are currently scattered in thousands of individual articles published in hundreds of journals often not open-access. Even more importantly, the computational analysis of the TTD data may prove useful to promote both the preclinical and clinical development of patient-tailored therapy based on the comprehensive (instead of piecewise) use of the available evidence.

### Data collection

The sources of the information input in the TTD are the PubMed, Medline, Embase, Cancerlit and Cochrane databases. Our literature search is aimed to identify scientific evidence about the relationship between:

- any molecule (each in a particular state, such as mutated, overxpressed, phosphorylated and so on) and the anti-melanoma efficacy of a therapeutic agent being used or being investigated for the treatment of melanoma (i.e., relationship of sensitivity/resistance);
- any molecule and the toxicity of any therapeutic agent being used or being investigated for the treatment of melanoma (i.e., relationship of toxicity);
- any molecule that - after modulation of its functional state by a “modifier” (e.g., inhibition by a drug) - can increase (or decrease) the efficacy a therapeutic agent being used or being investigated for the treatment of melanoma (i.e., relationship of synergism/antagonism).

Only original full-length articles are taken into consideration, so to guarantee that the data collected in the TTD are supported by research works whose methods, results and conclusions are fully reported in a manuscript that has passed through a standard peer-review process.

At the time of writing, over 1,200 records (ie, database rows) have been created, which cover more than 50% of the relevant literature published between January 2000 and January 2010, while for previous years the coverage is currently less than 50%. Our commitment is to complete the literature search back to January 1990 over the next 12 months.

Our search is systematic, that is, no key word other than “melanoma” is utilized, the only restriction being the English language. Accordingly, any type of study (i.e., preclinical/clinical, human/animal, in vitro/in vivo) regarding any type of melanoma (i.e., cutaneous, mucosal, uveal) is allowed to contribute to the content of the database.

### Data organization

Information is extracted from each retrieved article according to the following driving principle: the Authors of each article describe their findings and virtually always come to a main conclusion, whether “positive” (e.g., a molecule in a specific state can favor tumor response to a given treatment), “negative” (e.g., a molecule in a specific state can oppose tumor response) or “null” (e.g., tumor response is unaffected by a given molecule in a specific state). In other words, each study sustains one targeted therapy hypothesis, whether positive (the relationship between molecule and drug is favorable for the patient), negative (unfavorable) or null (unimportant, not influential).

Data are organized in rows and columns using a Microsoft Excel file. Each row contains the main data representing the targeted therapy hypothesis made by the Authors of a given article. Each column contains one type of data according to a standardized format.

The following 15 columns compose the database:

1. *ID*: this is a unique number identifying each record (that is, each row of the database).
2. *Source*: this indicates the tissue/cell type where the molecule under investigation (see next column) is expressed/present. For instance: somatic mutations of BRAF are investigated in melanoma specimens, polymorphisms of genes involved in drug metabolism can be studied in any patient's nucleated cell, and expression of cytokine receptors can be assessed in immune cells.
3. *Molecule*: this is the name of the molecule under investigation as a tumor-specific target, or as a biomarker of sensitivity/toxicity of melanoma/patient to therapeutic agents. The molecule's name is generally that reported by the Authors of the corresponding article.
4. *Alias (molecule)*: since molecules often have multiple names, aliases are reported in this column in order to clarify molecules' identity. Aliases are chosen on the basis of international databases such as HUGO (http://www.genenames.org) and Uniprot (http://www.uniprot.org).
5. *State (molecule)*: this refers to the condition (e.g., mutated, overexpressed, phosphorylated) under which the molecule exerts the biological activity related to the targeted therapy hypothesis reported in the article. For instance, the expression “mut V600E” for the protein BRAF refers to its V600E mutation (as opposed to the wild type protein or any other mutational status).
6. *Modifier*: this refers to any drug or drug-like compound or laboratory method that can modulate the biological function of a molecule of interest so to interfere with the efficacy of a therapeutic agent. For instance, a small molecule inhibitor can decrease the activity of a target molecule, which may ultimately affect the efficacy of an anticancer drug; likewise, technology based on RNA interference (e.g. small interfering RNA) can downregulate the expression of a gene of interest which may ultimately impact on the melanoma sensitivity to a given treatment.
7. *Alias (modifier)*: since modifiers often have multiple names, aliases can be found in this column in order to facilitate their identification.
8. *Relationship*: this column reports the hypothesized relationship between the molecule of interest and the corresponding treatment/drug (see “Drug” column). Three main types of relationships are considered: A) **Efficacy**: the molecule under investigation can be associated with either sensitivity or resistance to a therapeutic agent; B) **Synergism**: the modulation of a molecule activity by a modifier (see “Modifier” column) can be associated with an increased (synergism) or decreased (antagonism) therapeutic activity of a given drug/treatment; C) **Toxicity**: the molecule under investigation can be associated with either increased or decreased toxicity of a given drug/treatment. Of course, all these associations can be reported to be absent. For the purpose of prompt identification, positive (i.e. with positive effects on anti-melanoma treatment), negative (i.e. with adverse effects) and null associations are highlighted with different colors (green, orange and blue, respectively).
9. *Drug (therapy)*: this is the drug (or more generally the treatment) whose effectiveness can be influenced (positively, negatively or not significantly) by the molecule listed in column “Molecule”. The drug's name generally is that reported by the Authors of the corresponding article.
10. *Alias (drug)*: since drugs often have multiple names, one alias of the drug of interest is often reported in this column in order to clarify its identity.
11. *Model*: this column reports the model used by the Authors to generate the hypothesis. Seven different models are considered:
  1. animal, in vitro (e.g., murine melanoma cell line)
  2. animal, in vivo (e.g., syngeneic murine melanoma model)
  3. human in vitro (e.g., human melanoma cell line)
  4. human xenograft (e.g., human melanoma xenogeneic model)
  5. clinical study/non-randomized clinical trial
  6. randomized controlled trial
  7. meta-analysis of clinical trials/studies

This order is dictated by the “distance” of the model from the human-in vivo condition, or - in other words - by the level of evidence of the published data. This order will play a key role in the “weight” assigned to each study, as described in detail later on.

1. *H (hypothesis)*: As above mentioned, each article can be classified according to the main conclusions of its Authors supporting a “positive” hypothesis (e.g., a molecule in a specific state can favor tumor response to a given treatment), “negative” hypothesis (e.g., a molecule in a specific state can oppose tumor response) or “null” hypothesis (e.g., tumor response is unaffected by a given molecule in a specific state). Following this principle, each record (row) of the TTD is assigned a value that identifies the corresponding hypothesis (+1, −1 or 0, respectively).
2. *Cases*: this is the number of cases (e.g., patients, animals, cell lines) examined. At present, this information is only available for clinical studies/trials (i.e., number of patients).
3. *Reference*: the citation of the source of information is reported.
4. *Notes*: additional information on the study results/features can be found in this column in order to facilitate the interpretation of the data reported in the previous columns. This information can help users understand whether or not the molecular condition described in the record applies to their research/clinical question.

The information found in the TTD regards cutaneous melanoma, except for drug toxicity data (which are independent of the tumor type). If the entry relates to uveal melanoma, this is specified at the beginning of the column “Notes” by the bolded expression “Uveal melanoma”. Therefore, should one be interested exclusively in targeted therapy for uveal melanoma, data must be ordered by column “Notes”: this way the information contained in this column is rearranged in the alphabetical order and data on uveal melanoma will appear towards the end of the database as a sequence of rows tagged by the expression “Uveal melanoma” written in the column “Notes”.

Likewise, information on specific subtypes of melanoma (e.g., acral lentiginous melanoma, mucosal melanoma) can be easily retrieved using the same method.

Information on gene polymorphisms and drug toxicity can derive from non-melanoma models, as specified in the “Notes” column in bold character.

## Results

### Synopsis of the evidence

As above mentioned, the goal of the TTD is to enable investigators to find targeted therapy related information organized in a standardized and computationally oriented fashion. Since data are collected in an Excel file, they can be ordered by each of the 15 columns and also by any combination of three columns is sequential order.

**Figure 1:**
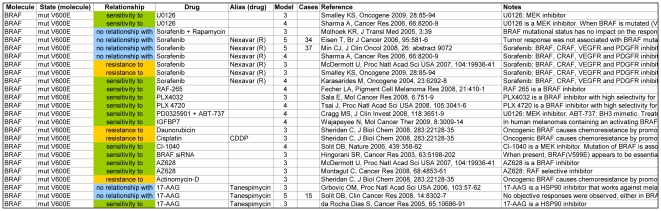
Example of evidence synopsis regarding the targeted therapy of melanoma, as obtained by searching the Targeted Therapy Database (TTD). The available evidence on the relationship between a molecule state (BRAF mutation V600E) and its effects on different therapeutic agents is shown. Due to space considerations, neither all columns nor all rows are displayed.

For instance, by sorting the database by “Molecule”, “State” and “Drug” (in this order), one can easily obtain for each molecule (and its state) the list of therapeutic agents whose efficacy is influenced by that molecule (in that particular state), as shown in **Figure 1**.

**Figure 2:**
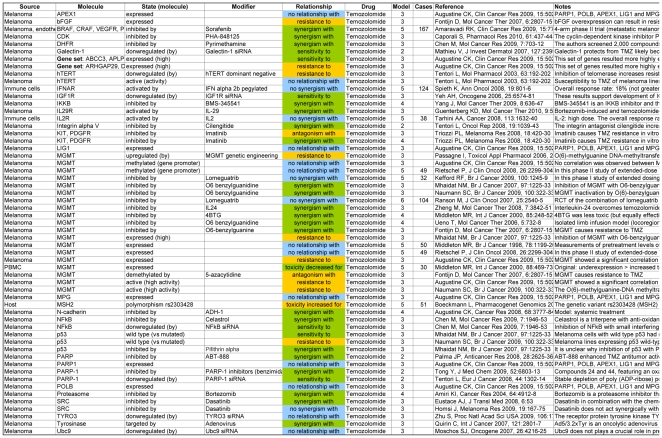
Example of evidence synopsis regarding the targeted therapy of melanoma, as obtained by searching the Targeted Therapy Database (TTD). The available evidence on the relationship between a drug (temozolomide) and the molecular determinants of its therapeutic effect is shown. Due to space considerations, neither all columns nor all rows are displayed.

On the other hand, by sorting the database by “Drug”, “Molecule” and “State” (in this order), one can easily obtain for each therapeutic agent the list of molecules (and their state) that can modulate its efficacy, as shown in **Figure 2**.

Likewise, by sorting the database by “Drug”, “Relationship” and “Modifier” (in this order), one can easily obtain for each therapeutic agent the list of compounds that can modulate its efficacy.

Obviously, many other searches can be performed by ordering the columns on the basis of a specific interest (e.g., evidence only from human models) or research question (e.g., “what gene polymorphisms affect the toxicity of cisplatin ?”).

### Summary of the evidence

One aim of the TTD is to allow researchers to conveniently summarize the available evidence on a given subject. This is an important feature because the scientific literature routinely poses the problem of multiple (sometime overwhelmingly numerous) inputs that often are not concordant (if not conflicting).

The standard way of making a quantitative review of the available scientific knowledge is performing a meta-analysis, which is considered the highest level of evidence in medicine, particularly when based on randomized controlled trials [51], [52], [53], [54], [55], [56]. The basic idea behind a meta-analysis is to calculate the weighted mean of the results reported by different studies regarding a particular subject; to this aim, the following key steps must be taken: 1) an effect measure (e.g., odds ratio, hazard ratio, relative risk, risk difference, mean, rate) common to all the studies must be identified; 2) the effect size (and its variance) must be extracted (or calculated) from each study; and then 3) the weighted mean of the effect sizes (overall effect) can be computed. From a therapeutic perspective, the overall effect quantifies the benefit (or the harm) of a given treatment, and the confidence interval (CI) represents the measure of uncertainty about its estimate (which in turn determines the statistical significance in terms of type I error, based on the predefined alpha level of significance).

In the light of these considerations, one can see that meta-analysis is *not* appropriate for summarizing the information contained in the TTD. In fact, the different effect measures adopted by the Authors to describe the results obtained in different models (ranging from animal in vitro models to randomized clinical trials) cannot be pooled together. Moreover, even if the effect measures were the same, different experimental models cannot be considered equally informative and reliable: obviously, human and in vivo models provide a higher level of evidence as compared to animal and in vitro models (provided that each study is equally well designed, performed and analyzed).

Therefore, the TTD cannot be exploited to calculate an overall effect size for a given therapeutic approach, which is why it does not record the effect sizes of the single studies.

What then is meant by “summary of the evidence” within the TTD ?

**Figure 3:**
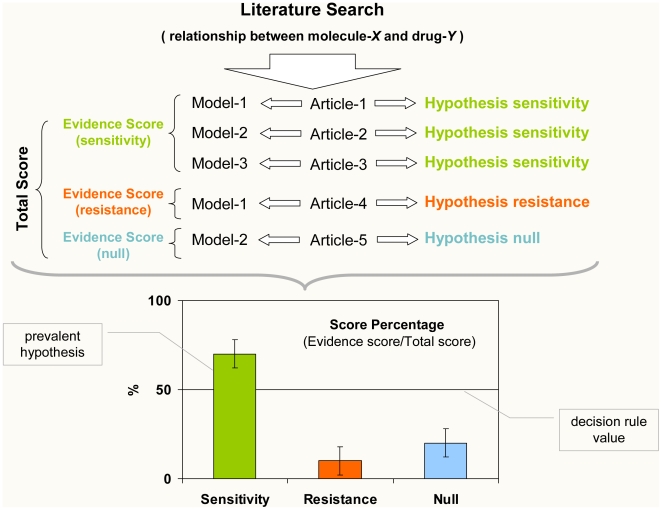
A scheme of the evidence score method to synthesize the literature evidence and identify prevalent hypotheses regarding the relationship of sensitivity/resistance between a given molecule (in a specific state) and a given drug. Each study is assigned an evidence score based on the experimental model used to generate the findings reported in each article In this example, 70% of the total score (that is, 70% of the published evidence rated according to the experimental model used to generate the findings reported in each article) supports the hypothesis that molecule-*X* (in a particular state, here not specified for the sake of simplicity) is associated with responsiveness to drug-*Y*. To be defined as “prevalent”, the hypothesis must be characterized by the fact that the lower bound of the 95% confidence interval of its score percentage does not cross the decision rule value (50%). The same method can be used to identify prevalent hypotheses regarding the relationship of toxicity and synergism (see text for more details).

As above mentioned, each study (which is represented by a row of the database) can be envisaged as a working hypothesis about a targeted therapy against melanoma. When more than one record (i.e., one row of the database) exists for a given hypothesis (e.g., BRAF mutation V600E modulates the efficacy of small molecule inhibitor sorafenib), we propose a score-based approach to make a summary of the available evidence. With this method we aim to identify the “prevalent” hypothesis, a process taking the following steps (see also **Figure 3**):

**1)** As reported in column “H (hypothesis)”, each record (i.e., each row of the database) is assigned one of the integer numbers “+1”, “−1” or “0”, based on the fact that it represents a piece of evidence in support of one of the three possible hypotheses (as expressed by the Authors of the corresponding manuscript):

1. *positive relationship* (green color in the “Relationship” column): the study supports the hypothesis that the molecule (e.g. BRAF) in a particular state (e.g. mutation V600E) is associated with increased efficacy of a drug, synergism between drugs or decreased toxicity of a drug. On the practical ground, a patient carrying this molecule (in this specific state) would benefit from the given treatment;
2. *negative relationship* (orange color): the study supports the hypothesis that the molecule can oppose the efficacy of the drug; a patient (tumor) carrying this molecule (in this specific state) would be refractory to the given treatment
3. *null relationship* (blue color) if the study supports the hypothesis that the molecule does not change the efficacy of the drug; knowing that a patient (tumor) carries this molecule (in this specific state) would be uninformative in terms of responsiveness to the treatment.

**2)** As reported in the “Model” column, each record is also assigned a score (**model score**), based on the experimental/clinical model used to generate the targeted therapy hypothesis. Clearly, the evidence coming from an in vitro study carried out with murine melanoma cell lines cannot have the same “weight” as the evidence derived - for instance - from a study performed in a human trial model. The closer the model to the in vivo human condition, the higher the level of evidence and thus the greater is the weight assigned to that study.

Within the frame of this arbitrary score, the proportion between the weights of “adjacent” models is fixed: in particular, the score of each model is twice that of the immediately precedent model. The starting score (model: animal, in vitro) was set to 6 because this is the smallest natural number that meets the decision rule below described (in case a single study based on such a model supported a given hypothesis).

The evidence score is then adjusted according to an additional weight (**size score**), which is based on the number of cases (e.g., patients, animals, cell lines) analyzed (“Cases” column): this way, studies describing results obtained from larger series are assigned a higher score.

The total **evidence score (ES** ***_i_*** **)** for each hypothesis *i* is computed according to the following formula:
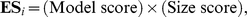
where Size score = *n*/10 (*n* is the sample size [e.g., number of patients enrolled] of the study under evaluation).

**3)** The percentage of the evidence score (**score percentage, SP**) in favor of each of the three above mentioned hypotheses (i.e., positive, negative, null) is simply defined as the proportion between the evidence score in favor of each hypothesis *i* and the sum of the evidence score of all hypotheses:
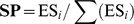



**4)** At this point, a decision rule must be applied to determine whether or not a prevalent hypothesis exists: we chose 50% (0.5) of the evidence score as the minimum value to define the prevalent hypothesis. In other words, if one of the three possible hypotheses (i.e., positive, negative, null) is associated with more than 50% of the available evidence score and the lower level of the 95% CI of this proportion does not cross this decision rule value, one can reasonably suppose this is the prevalent hypothesis in the scientific literature.

The 95% CI of the score percentage (SP) can be calculated according to the Agresti-Coull formula (which provides a substantial improvement over the widely used Wald method especially for proportion values near 0 and 1 and for small sample sizes, as it can occur in the TTD):
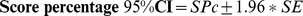
where:

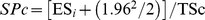
, that is the score percentage (SP) corrected according to the Agresti-Coull method

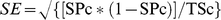
, that is the standard error of SPc

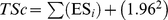
, that is the total evidence score (supporting any given hypothesis *i*) corrected according to the Agresti-Coull method.

A formal comparison between a given score percentage (SP) and the 50% (0.5) decision rule value can be made using a Z-test, according to the following formula:
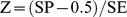
where:

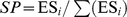

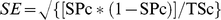

For a two-tailed test, the P-value is given by:
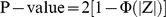
where Φ (|Z|) = standard normal cumulative distribution.

Of course, the decision rule value (0.5) can be shifted up or down to make it more or less stringent respectively, thus rendering more or less conservative the conclusion regarding the relationship between the patient's profile and the response to treatment.

If none of the three hypotheses meets the decision rule, we can reasonably suppose that there is no prevalent hypothesis, that is, there is not enough evidence to link a given molecule (in a particular state) to the efficacy/synergism/toxicity of a given drug.

**5)** Once we know that there is enough evidence to support the hypothesis that no relationship exists between a molecule and a drug, or that not enough evidence exists to support any hypothesis on this relationship, this molecule is eliminated from the list of molecules useful to predict drug responsiveness. Importantly, this is not a definitive elimination, because new data will likely be published on this relationship and thus the result of the summary can change at any time. Since the TTD is routinely updated, the selection of relevant molecules is a dynamic process that can provide different results over time as the scientific knowledge grows.

**6)** If the summary of evidence is instead in favor of the hypothesis that a molecule (in a particular state) can modulate (either positively or negatively) the activity of a treatment, then that molecule is added to the list of molecules potentially useful (i.e., informative) to predict the responsiveness to the treatment.

*To provide readers with a working example of the computations here described, the above algorithm is fully implemented in the TTD spreadsheet entitled “Summary of Evidence” (available as an open-access file in the MMMP website).*

### Drug ranking system

Once a list of molecules for which “consistent” evidence is available in favor of their role in predicting the responsiveness (or refractoriness) to a specified therapeutic agent, as assessed by means of the above described summary of the evidence, one might be willing to test the relevant biospecimens from a given patient for these molecules and match the patient's molecular profile with the currently available evidence on targeted therapy.

This opens the avenue to the use of the already available scientific knowledge for generating hypothesis of personalized treatment based on the fundamental principle of molecular medicine: to use the patient (disease) molecular profile for designing the treatment most effective and least toxic.

Before entering the technical details, one crucial issue must be clearly addressed. The TTD has exclusively research purposes, and thus neither the information nor the analytical models included in this database should be used for the clinical decision making process by any means. In fact, this way of summarizing the evidence across (sometime very) different models has never been reported before and thus it requires adequate validation before it can be considered reliable on the clinical ground.

**Figure 4:**
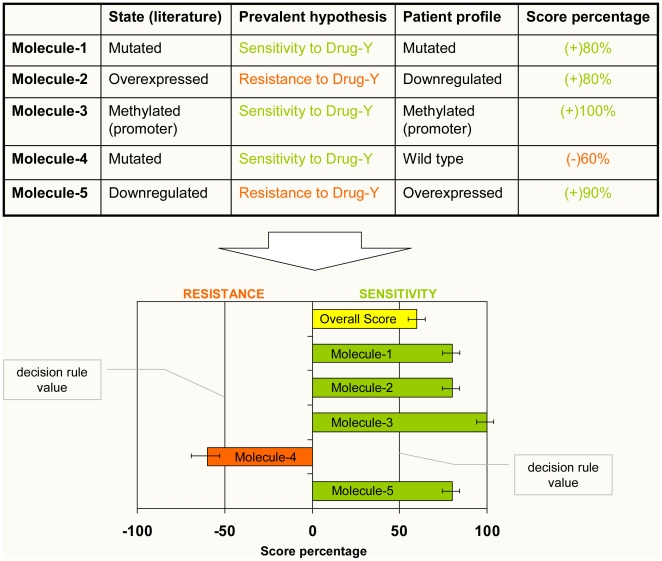
A scheme of the drug ranking system to match the patient's molecular profile with the available scientific evidence regarding the relationship of sensitivity/resistance between a set of molecules (each in a specific state) and a given drug. After identifying the prevalent hypothesis (along with its score percentage) for each molecule according to the evidence score method (see text and Figure 3 for more details), the same molecules (and their state) are tested in the tumor of a patient. Each molecule is said to be concordant (positive sign) or discordant (negative sign) according to whether the molecule state found in the patient's tumor is identical or opposite to the state reported in the literature, respectively. Then, a weighted mean of the score percentages is calculated to obtain the overall score for the patient. In this example, the overall score indicates that on average 60% of the available evidence (that is, 60% of the published evidence rated according to the experimental model used to generate the findings reported in each article) is in favor of the hypothesis that the patient's molecular profile is associated with responsiveness to drug-*Y*. To be defined as “sensitive” (or “resistant”), a molecular profile must be characterized by an overall score with a lower bound of its 95% confidence interval that does not cross the decision rule value (+50% or −50%, respectively). The same method can be used to assess whether the available evidence supports the hypothesis that a molecular profile is associated with higher/lower toxicity for a given drug-*Y* (see text for more details).

With this important caveat in mind, we propose to take the following steps in order to match the patient's molecular profile with the current evidence on targeted therapy (see also **Figure 4**):

**1)** Using the above described score-based system, the informative molecules (each along with a particular state of expression/function) are extracted from the TTD along with their score percentage (SP) and 95% CI. *Each SP can be viewed as a measure of strength of the hypothesis sustaining the relationship between the molecule and the drug efficacy (toxicity, synergism) based on the available literature as rated by the evidence score above described.*

**2)** Score percentages (SP) of molecules associated with sensitivity to treatment are initially assigned a “+” sign (e.g. BRAF mutation V600E increases the efficacy of drug Sorafenib), whereas molecules associated with resistance to treatment are assigned a “−” sign (e.g. BRAF mutation V600E decreases the efficacy of drug Sorafenib). Then, the concordance (or discordance) between the molecular state of the prevalent hypothesis and that of the patient (tumor) must be assessed. In particular, the sign of the SP will be left unchanged if the patient carries the same molecular state as that of the SP (e.g. BRAF mutation V600E); in contrast, if the patient carries the “opposite” molecular state (e.g. BRAF wild type), the SP will be assigned the opposite sign.

**3)** At this point, an **overall score (OS)** can be calculated as the weighted average of the score percentage calculated for each informative molecule. The OS and its confidence interval can be calculated using the inverse variance method as follows:
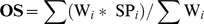
And
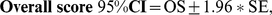
where:

*SP_i_* : score percentage of the prevalent hypothesis calculated for each molecule (in a specific state) for which the patient (cancer) has been tested
*W_i_* = 1/V*_i_*, the weight assigned to each molecule based on the variance of the SP
*V_i_* = SPc * (1−SPc), i.e. the variance of the SP calculated for each molecule (see above)
*SE* = standard error = √ (OV)
*OV* = overall variance = 1/Σ W_i_

The interpretation of the resulting score obviously depends upon the decision rule one adopts. Using the 50% decision rule (as we suggested for the summary of the evidence), two outcomes can occur:

- if the overall score for a given patient is greater than 50% (0.5) and its 95% CI does not cross the 50% decision rule value, one can reasonably conclude that the available evidence supports the hypothesis that this specific profile is associated with sensitivity (or resistance, depending on the “direction” of the overall score) to the treatment under evaluation;
- if the overall score for a given patient either is lower than (or equal to) 50% (0.5) or its 95% CI crosses the 50% decision rule value, one can reasonably conclude that there is not enough evidence linking this specific profile to the responsiveness (or refractoriness) to the treatment under evaluation.

A formal comparison between the calculated overall score (OS) and the 50% (0.5) decision rule value can be made using a Z-test, according to the following formula:
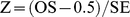
where OS and SE are defined as above reported. For a two-tailed test, the P-value is given by:
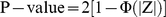
where Φ (|Z|) = standard normal cumulative distribution.

*To provide readers with a working example of the computations here described, the above algorithm is fully implemented in the TTD spreadsheet entitled “Profile Matching” (available as an open-access file in the MMMP website).*

Of course, the decision rule value (0.5) can be shifted up or down to make it more or less stringent respectively, thus rendering more or less conservative the conclusion regarding the relationship between the patient's profile and the response to treatment.

In this regard, we plan to validate the predictions of our model by fitting logistic regression analysis to the scores generated by the TTD. This is a standard approach for binary outcome prediction models (responder vs. non-responder) and has several useful features: 1) it allows to adjust for confounding factors (e.g., age, gender, clinical setting, previous treatments) and even for the creation of a multivariable prediction model using the logistic regression linear predictor as a composite prediction score (which would allow to synergistically exploit the predictive power of multiple covariates); 2) predictive accuracy can be defined in terms of discrimination and calibration by means of dedicated statistics (e.g., Brier score and its decomposition); 3) Receiver Operating Characteristic (ROC) curve analysis can help choose the optimal score trade off value to define responders (currently set to 50%).

**4)** If the above procedure is performed for more than one treatment (i.e., the patient's molecular profile is matched with more than one therapeutic agent), it is also possible to create a drug rank based on the overall score obtained for each drug as above outlined. A formal comparison between two overall scores (e.g., OS_a_ and OS_b_) relative to the matching of the patient's profile with drug *A* and drug *B* can be computed using a Z-test, according to the following formula:
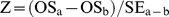
where:

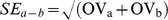

*OV_a_* : variance of the overall score for the matching of patient's profile with drug *A*
*OV_b_* : variance of the overall score for the matching of patient's profile with drug *B*

For a two-tailed test, the P-value can be calculated using the following formula:
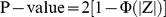
where Φ (|Z|) = standard normal cumulative distribution.

Of course, the same procedure can be used to match the patient's molecular profile with the available evidence regarding drug/treatment toxicity.

## Discussion

The Targeted Therapy Database is the first publicly available repository that provides investigators with a searchable and computation-compatible collection of the scientific evidence regarding the targeted therapy of melanoma. Users can query the database to easily obtain standardized information about the molecular determinants of sensitivity or resistance of melanoma to a given treatment, the compounds that can synergize with a given treatment, as well as the molecular determinants of toxicity of a given treatment.

This information can be utilized to quickly ascertain the most studied as well as the emerging therapeutic strategies, along with the models where they have been tested and the results yielded so far.

Using the above presented model based on the evidence score, these data can also be exploited to identify prevalent therapeutic hypotheses, which is especially helpful when conflicting results are reported in the literature. As above explained, although our model cannot quantify the therapeutic benefit of a given targeted therapy, it can be used to discern trends in the available evidence, pinpointing the most promising approaches based on the amount of literature (rated according to the scoring method described above) in favor of each therapeutic hypothesis.

Finally, this archive - along with the algorithm we have proposed - can be utilized to match the patient's molecular profile with the available literature and thus to hypothesize patient-specific drug sensitivity toxicity or synergism based on the scientific evidence supporting each type of relationship for each of the molecules investigated.

We chose melanoma because this tumor paradigmatically represents the urgency of providing patients with better treatments: in fact, no current drug regimen significantly impacts on the clinical course of this disease in the metastatic setting. Under these unfortunate circumstances, any therapeutic choice based on the available evidence (even without clinical proof of efficacy of such a strategy) would appear more rational than offering patients no options at all. However, since the drug ranking system described above is based on a theoretical model, it should only be used to generate hypotheses, not to make clinical decisions. In other words, at the moment the findings obtained with our model should only be used *a posteriori* (after the patients has been treated with a regimen chosen independently of the model results) in order to determine the actual performance of the model itself. Only this validation of the model on the clinical ground will enable us to verify whether our theoretical computations are accurate enough to be clinically valuable, and thus to propose the implementation of the model in the routine setting for choosing the therapeutic regimen most likely to benefit individual patients.

Despite its intrinsic limitations (e.g., the score is arbitrary, the literature coverage is incomplete and thus many hypothesis are based on few or even single original articles), this model is - to the best of our knowledge - the first attempt to directly apply the enormous amount of data accumulated by the scientific community in the field of personalized medicine. This translational approach has the undeniable advantage of making the most of the scientific production by using it comprehensively, without wasting any evidence. This can be envisaged as an effort to deal with the general problem that the biomedical community produces more data than those utilized for clinical purposes. The actual impossibility of testing each preclinical hypothesis in the clinical setting represents undoubtedly a waste of potentially useful information: this “abandoned” information could be “rescued” by taking it into consideration through the model we propose for the evidence-based design of further research, both preclinical and clinical. Should the clinical validation of this drug ranking system demonstrate that it is reliable, the TTD could be utilized as a template to develop similar repositories dedicated to any tumor and more generally to any disease.

On the other hand, it should be clearly noted that scoring the hypotheses reported in the literature as we propose to do here cannot replace the standard rules of research, including clinical phases of treatment evaluation and formal meta-analysis of therapeutic interventions. The model we presented can only speed up the identification of the most promising hypotheses of targeted therapy by making an unprecedented comprehensive use of the available evidence based on two principles: 1) any information is potentially useful, independently of the experimental model that has generated it, provided that different “weights” are assigned to different models in order to reflect the difference in reliability; 2) disease's outcomes virtually always depend upon molecular combinations, which calls for the simultaneous use of information about all the molecules so far investigated, which should maximize the likelihood of successfully drive targeted therapies.

As the available and eligible data are added to the TTD, we will be able to make predictions more and more reliable because they will be based on more information. In particular this will minimize the risk of publication bias because some positive/significant molecular associations published in the first place will be “balanced” by negative/non significant findings. We note that - in analogy to standard meta-analysis - the greater the number of studies considered the smaller the variance of the overall effect; in our case, the smaller the sampling error the more accurate the prediction. Furthermore, the growing information will enable investigators to make setting specific predictions thanks to the flexibility of the TTD: in fact, its format allows to insert more columns (e.g., a new one could be dedicated to distinguish data obtained in the primary tumor or metastatic setting) at any time. Then our model can still be applied as above described because the user can simply sort the database by the new column (e.g., primary vs. metastatic) and use only the relevant information (e.g., data from primary or metastatic setting) based on the clinical question to be addressed.

Finally, we would like to underscore that this kind of project can succeed only if the scientific community participates in the effort of improving the model we have proposed. This can be realized in several ways, such as: A) by giving notice of relevant articles not yet included in the TTD, which will maximize the literature coverage of the database and thus will ultimately increase the reliability of the analyses performed; B) by proposing new algorithms improving the exploitation of the information contained in the database; C) most importantly, by testing the hypotheses generated by the TTD analyses both in the preclinical and clinical setting.

Overall, putting together the pieces of a “disease puzzle” is becoming increasingly difficult due to the continuous and growing flow of information that no single mind can keep up with: we therefore propose the TTD (and the associated model for drug ranking) as a tool for the synopsis and synthesis of the scientific hypotheses with the aim of favoring the rational design of both preclinical and clinical research.

The commitment of the MMMP Team (the core of basic researchers and clinical investigators taking care of the scientific content of the MMMP website) is not only to keep the TTD regularly updated but also to carefully take into consideration suggestions, criticisms and contributions from the scientific community.

We strongly believe that the bidirectional exchange of information (from the database to the user and vice versa) represents the most efficient way of gathering and exploiting scientific data on a specific disease: in fact, if every researcher spent just a small amount of time to share his/her knowledge to keep up-to-date the TTD or any other similar project, the pace of discovery of more effective anticancer strategies would be greatly increased.
